# miR-194-3p regulates epithelial-mesenchymal transition in embryonic epicardial cells via p120/β-catenin signaling: miR-194-3p regulates EMT via p120/β-catenin

**DOI:** 10.3724/abbs.2024051

**Published:** 2024-04-26

**Authors:** Tianhua Xiong, Dinghui Wang, Huiping Yang, Bin Liu, Yingrui Li, Wenlong Yu, Jing Wang, Qiang She

**Affiliations:** Department of Cardiology The Second Affiliated Hospital of Chongqing Medical University Chongqing 400010 China

**Keywords:** embryonic epicardial cells, epithelial-mesenchymal transition, development, miR-194-3p, p120-catenin

## Abstract

The epicardium is integral to cardiac development and facilitates endogenous heart regeneration and repair. While miR-194-3p is associated with cellular migration and invasion, its impact on epicardial cells remains uncharted. In this work we use gain-of-function and loss-of-function methodologies to investigate the function of miR-194-3p in cardiac development. We culture embryonic epicardial cells *in vitro* and subject them to transforming growth factor β (TGF-β) treatment to induce epithelial-mesenchymal transition (EMT) and monitor miR-194-3p expression. In addition, the effects of miR-194-3p mimics and inhibitors on epicardial cell development and changes in EMT are investigated. To validate the binding targets of miR-194-3p and its ability to recover the target gene-phenotype, we produce a mutant vector *p120-catenin*-3′UTR-MUT. In epicardial cells, TGF-β-induced EMT results in a notable overexpression of miR-194-3p. The administration of miR-194-3p mimics promotes EMT, which is correlated with elevated levels of mesenchymal markers. Conversely, miR-194-3p inhibitor attenuates EMT. Further investigations reveal a negative correlation between miR-194-3p and p120-catenin, which influences β-catenin level in the cell adhesion pathway. The suppression of EMT caused by the miR-194-3p inhibitor is balanced by silencing of *p120-catenin*. In conclusion, miR-194-3p directly targets p120-catenin and modulates its expression, which in turn alters β-catenin expression, critically influencing the EMT process in the embryonic epicardial cells via the cell adhesion mechanism.

## Introduction

Cardiovascular illnesses continue to be one of the most serious global health threats, endangering the lives of millions of people. Central to the heart’s function is the epicardium, an integral component that plays an essential role in cardiac development and endogenous regenerative processes [1]. A single layer of precursor epicardial cells develops into smooth muscle and fibroblasts, which in turn formcardiac endothelial cells and interstitial cells [ 2, 3]. By embryonic day (E) 9.5 in mice, precursor epicardial cells attach to the myocardium and crease in the epithelial layer to form a single cell layer that envelops the entire myocardium [4]. By E11.5, the heart is fully covered by epicardial cells, exhibiting a cuboidal epithelial phenotype [5]. Membrane adhesion molecules such as β-catenin and E-cadherin are expressed to preserve the integrity and top-to-base polarity of the epicardial cell layer [6]. Numerous genes encoding transcription factors, such as Wilms tumor 1 (Wt1) [7], transcription factor 21 (Tcf21) [8], and T-box transcription factor 18 (Tbx18) [9], are expressed in the epicardium.

Epithelial-mesenchymal transition (EMT), which occurs as the epicardium differentiates, is followed by stratification, which results in the formation of cells derived from the epicardium that span the gap between the epicardium and myocardium. These cells form the sub-epicardial mesenchyme and infiltrate the myocardium, where they evolve into supporting cells of the heart [ 10‒ 13]. TGF-β is among the most extensively studied upstream regulatory molecules for EMT and extracellular matrix (ECM) production [14]. TGF-β1–3 isoforms are expressed in the epicardium of mice at E12.5, and they bind to the same receptor complex, collectively inducing epithelial cell EMT [ 15‒ 18]. The transition of primary epithelial tissue into highly motile mesenchymal cells is a characteristic feature of the development of multicellular organisms. It promotes the growth and development of organisms by enabling epithelial cells to migrate inside the ECM and build tissues at particular sites [11]. Notably, the molecular mechanisms underlying the differentiation of epicardium-derived cells into distinct cell types during cardiac development remain elusive. Interestingly, adult epicardial cells are mostly quiescent, but when they are subjected to damage, they become active and aid in repair by re-expressing the adult heart’s developmental programs [19].

MicroRNAs, comprising 19–25 nucleotides, constitute a vital subset of small noncoding RNAs pivotal in post-transcriptional regulation of gene expression by targeting the 3′UTR of coding RNAs, resulting in the inhibition of protein translation and facilitation of mRNA degradation [20]. The essential involvement of miR-194-3p in cellular migratory and invasive activities has been shown in recent scientific reports [ 21‒ 23]. However, the precise modulatory role of miR-194-3p in the EMT of the epicardium during cardiac embryogenesis still needs to be addressed. Notably, while many *in vitro* models have been established to investigate epicardial EMT [ 24, 25], the regulatory orchestration of microRNAs, especially that of miR-194-3p, in this physiological transition has yet to be explored. Based on our research, we propose that miR-194-3p plays a crucial role in mediating epicardial EMT, possibly by influencing the cell adhesion signaling pathway.

At the forefront of this research gap, our work aimed to clarify the effects of miR-194-3p on cardiac development by analyzing the molecular mechanisms underlying its modulation of epicardial cell EMT using cultured embryonic epicardial cells.

## Materials and Methods

### Animals

C57BL/6J mice (males and females, 8 weeks old, weight: 20–25 g each) were provided by Chongqing Medical University Animal Breeding Center (Chongqing, China) and were kept under ideal conditions (humidity 40%±10%, temperature 23±2°C, and 12 h light-dark cycle). All the breeding and experimental procedures were conducted in accordance with protocols approved by the Animal Protection and Use Committee of Chongqing Medical University. All animal studies adhered to the ARRIVE guidelines [26]. C57BL/6J female and male mice were co-housed at a ratio of 1:2 or 1:3 starting at 8 p.m. for mating. When a vaginal plug was found in the female mice by 8 a.m. on the next day, the embryonic age was recorded as E0.5.

### *In vitro* culture of embryonic epicardial cells and primary neonatal mouse cardiomyocytes

Sterilized 1% gelatin (BioFroxx, Einhausen, Germany) was applied to 12-well plates, and the plates were left to air dry. For immunofluorescence samples, a 14 mm×14 mm coverslip was placed in the plate prior to gelatin coating. Embryonic mouse hearts were harvested at E12.5, and the ventricles were transferred to a pre-cooled DMEM medium (Gibco, Waltham, USA). These ventricles were then precisely positioned in the gelatin-coated plate and covered with a coverslip. After addition of complete cell culture medium, the plates were incubated at 37°C with 5% CO _2_. After 1–2 days, the emergence of cobblestone-like epicardial cells was observed around the ventricles. The coverslips were subsequently rotated, the ventricles were removed, and the wells were washed with PBS before addition of fresh culture medium. The medium was refreshed every two days, and cell morphology was continuously monitored.

Neonatal cardiomyocytes were isolated from 1-day-old C57BL/6J mice. The mice were anesthetized with a 10-min cold exposure, then euthanized and their bodies were disinfected with 75% ethanol. The heart was exposed by an incision along the left side of the sternum and subsequently dissected in a sterile environment into an ice-cold enzyme-free dissociation solution. Atria and large vessels were removed over sterile gauze, and ventricular tissue was minced before being subjected to enzymatic digestion in a 37°C water bath with periodic agitation. The digested tissue was sequentially centrifuged (80 *g*, 37°C, and 5 min) in the presence of fetal bovine serum, and the supernatants were discarded. After multiple digestion steps, cells from all collections were pooled, filtered through a 40-μm mesh and centrifuged (80 *g*, 37°C, and 5 min). The resulting cell pellet was resuspended in fresh culture medium and allowed to adhere for 60 min. The non-adherent cells were collected and seeded into 6-well plates that had been coated with 1% gelatin under aseptic conditions. Cells were cultured at 37°C with 5% CO _2_, with medium changed every two days.

### Cell treatment

Primary epicardial cells isolated from embryos were cultured in DMEM supplemented with 10% fetal bovine serum (FBS) (Gibco), 100 U/mL penicillin (Beyotime Biotechnology, Shanghai, China), and 100 μg/mL streptomycin (Beyotime Biotechnology) in a humidified atmosphere at 37°C with 5% CO _2_. For TGF-β treatment experiments, after two days in culture, cells were exposed to fresh complete culture medium containing TGF-β1 recombinant protein (Abcam, Cambridge, UK) at a concentration of 10 ng/mL. After 48 h of treatment, cellular changes were observed to assess TGF-β-induced differentiation.

### Immunocytochemistry assay

The cells were washed with phosphate-buffered saline (PBS) three times, fixed in 4% paraformaldehyde (Boster Biological Technology Co., Ltd, Wuhan, China) for 10 min, and permeabilized using 0.25% Triton X-100 (Solarbio Science & Technology Co., Ltd, Beijing, China) for 10 min. After being blocked with 10% goat serum solution (Beyotime Biotechnology) for 30 min, they were incubated with the primary antibody (1:100 dilution) overnight at 4°C. The antibodies used included anti-Tbx18 (Abcam), anti-Wilms Tumor Protein antibody (Abcam), anti-Vimentin (Cell Signaling Technology, Danvers, USA) and anti-PDGFRα (Cell Signaling Technology). After another wash with PBS, cells were exposed to goat anti-rabbit IgG H&L-FITC (Abcam) or goat anti-mouse IgG H&L-TRITC (Abcam) secondary antibody (1:100 dilution) at 37°C for 45 min. Nuclear staining was performed with 4′,6-diamidino-2-phenylindole (DAPI; Beyotime Biotechnology) for 5 min. Finally, anti-fade reagents (Beyotime Biotechnology) were used to mount the specimens, and mages were captured with a confocal fluorescence microscope (Nikon, Tokyo, Japan).

### Transfection and dual-luciferase reporter assay

For miRNA and siRNA transfection, RFect ^PM^ primary cell small nucleic acid transfection reagent (BaiDai Biotech, Changzhou, China) was utilized. A mixture of RFect ^PM^ reagent and the desired miR-194 mimic (or inhibitor) or *p120* siRNA (sense strand sequence: 5′-GAGGUGCCGCCUGAUCAGUAC-3′, antisense strand sequence: 5′-ACUGAUCAGGCGGCACCUCUU-3′) diluted in Opti-MEM medium (Gibco) was prepared. After incubation at room temperature for a specific period, the mixture was added to the cells. Following a brief incubation period, the medium was then replaced by a fresh complete medium. Subsequent to transfection, cells in a 96-well plate were cotransfected with a dual-luciferase reporter vector containing the 3′UTR of the target gene harboring the putative miRNA binding site, along with the respective miR-194 mimic (guide strand sequence: 5′-CCAGUGGAGCUGCUGUUACUUC-3′, passenger strand sequence: 5′-GAAGUAACAGCAGCUCCACUGG-3′)miR-194 mimic NC (guide strand sequence: 5′-UUCUCCGAACGUGUCACGU-3′, passenger strand sequence: 5′-ACGUGACACGUUCGGAGAA-3′), miR-194 inhibitor (sequence: 5′-GAACAAGCAGCUCCACUGG-3′), or miR-194 inhibitor NC (sequence: 5′-CAGUACUUUUGUGUAGUACAA-3′). For normalization, reporter constructs containing a mutated 3′UTR miRNA binding site were used as controls. Approximately 48 h after transfection, the cells were lysed, and the luciferase activity was assessed using a Dual-Luciferase Reporter Assay System (Sangon Biotech, Shanghai, China). Firefly luciferase readings were standardized against Renilla luciferase activity.

### Wound healing assay

Embryonic epicardial cells were transfected with miRNA mimic or inhibitor and incubated for 48 h. These cells were cultured in 12-well plates. A straight scratch wound was created in the center of each well using a sterile 200-μL pipette tip. After removal of cell debris by gentle washing with PBS, the remaining cells in the plate were cultured in fresh medium. The wound was photographed at time intervals of 0, 4, 8, and 12 h using an inverted microscope (Nikon). The initial wound width and the width at each time point were compared to calculate the migration rate.

### Western blot analysis

Cells were lysed in radioimmunoprecipitation assay (RIPA) buffer (Beyotime Biotechnology) supplemented with protease and phosphatase inhibitors. The total protein concentration was determined using the Bicinchoninic Acid (BCA) Protein Assay Kit (Beyotime Biotechnology). Equal amounts of protein from each sample were separated by 10% SDS-PAGE and subsequently electrotransferred onto a polyvinylidene difluoride (PVDF) membrane (Bio-Rad Laboratories, Inc., Hercules, USA). The membrane was blocked for 1 h at room temperature with 5% non-fat milk in TBST (Tris-buffered saline with 0.1% Tween 20), and then incubated overnight at 4°C with specific primary antibodies diluted in blocking buffer. The antibodies used included anti-delta 1 catenin (Abcam), anti-E-cadherin (Cell Signaling Technology), anti-vimentin (Cell Signaling Technology), anti-PDGFRα (Cell Signaling Technology), and anti-β-actin (Cell Signaling Technology). Membranes were washed three times with TBST and then incubated with the corresponding horseradish peroxidase-conjugated anti-rabbit IgG H&L secondary antibody (Abcam) for 1 h at room temperature. Following three times wash with TBST, protein bands were visualized using Omni-ECL ^TM^ Enhanced Pico Light Chemiluminescence Kit (Epizyme Biotech, Shanghai, China) and band intensities were measured by densitometry using β-actin as a loading control.

### Quantitative real-time PCR (qRT-PCR) analysis

Total RNA was extracted from primary embryonic epicardial cells using the TRIzol (TM) Reagent (Thermo Fisher Scientific, Waltham, USA). RNA (1 μg) from each sample was reverse transcribed into cDNA using the HiScript II Q RT SuperMix for qPCR (Vazyme Biotech Co., Ltd, Nanjing, China), and qPCR was performed using the HiPlex Robustic SYBR Green Mix (Nuhigh Biotechnologies Co., Ltd, Suzhou, China) on a 7900HT Fast Real-Time PCR System (Applied Biosystems, Foster City, USA). The expression levels of the mRNAs and miRNAs were normalized to those of the endogenous reference genes *GAPDH* and *U6*, respectively, and relative expression levels were determined using the 2 ^–ΔΔCt^ method. The primer sequences are listed in Table 1.

**Table:** **Table 1** Sequences of primers used in qRT-PCR

Gene	Primer sequence (5′→3′)
*Tbx18*	Forward	CAGCTGACTATTCGGCCTGT
	Reverse	GAGTCCTAGGTGGGGCAAAG
*E-cadherin*	Forward	CAGGTCTCCTCATGGCTTTGC
	Reverse	CTTCCGAAAAGAAGGCTGTCC
*N-cadherin*	Forward	GTGGAGGCTTCTGGTGAAATTG
	Reverse	TCCTTCGTGCACATCCTTCG
*Vimentin*	Forward	CTCCTACCGCAGGATGTTCG
	Reverse	CGTGTGGACGTGGTCACATA
*PDGFRα*	Forward	AGTGGCTACATCATCCCCCT
	Reverse	CCGAAGTCTGTGAGCTGTGT
*Snail*	Forward	CCATTCTCCTGCTCCCACT
	Reverse	CCTGGCACTGGTATCTCTTCA
*Slug*	Forward	AGAAGCCCAACTACAGCGAA
	Reverse	ATAGGGCTGTATGCTCCCGA
*α-SMA*	Forward	GGGAGTAATGGTTGGAATGG
	Reverse	GGTGATGATGCCGTGTTCTA
*Fn1*	Forward	ATGTGGACCCCTCCTGATAGT
	Reverse	GCCCAGTGATTTCAGCAAAGG
*cTnT*	Forward	AGCCCACATGCCTGCTTAAA
	Reverse	TCTGAACAGGGACTGCACAC
*Kras*	Forward	CAAGAGCGCCTTGACGATACA
	Reverse	CCAAGAGACAGGTTTCTCCATC
*Camk2b*	Forward	TGATGTCCTGAGCTTGGTGAG
	Reverse	GGGGGCTAATGGGAACTGG
*p120*	Forward	GTGGAAACCTACACCGAGGAG
	Reverse	CGTCTAGTGGTCCCATCATCTG
*GAPDH*	Forward	TGGCCTCCAAGGAGTAAGAAAC
	Reverse	GGCCTCTCTCTTGCTCTCAGTATC

### Gene ontology (GO) analysis

Following identification, the anticipated target genes were subjected to a thorough Gene Ontology (GO) analysis to understand their putative biological roles and mechanisms. For the annotation and functional enrichment analysis, we employed Metascape [27], an integrated tool optimized for deciphering multifaceted biological insights.

### Protein-protein interaction (PPI) network analysis

The nodes in the PPI network represent proteins, while associations are represented by the connections between nodes. Since proteins are the products of gene expression, the connections between protein molecules can determine the relationships between molecules, the relationships between genes can be understood, and the core regulatory genes can be mined. PPI was analyzed by STRING ( https://cn.string-db.org/), and a PPI network was constructed by Cytoscape, an open-source software platform for visualizing complex networks and integrating these networks with any attribute data. Cytoscape was used to analyze further and visualize the PPI network.

### Statistical analysis

Each experiment was performed at least in triplicate. Data are presented as the mean±standard deviation (SD). Student’s *t* test (Mann-Whitney U test) was used for comparison between two groups, and analysis of variance (ANOVA) was used for compariosn amomg several groups. The statistical analyses were performed using GraphPad Prism v9.0 software (GraphPad Software, Inc., La Jolla, USA) and the R package (version 4.2.1). *P*<0.05 was considered statistically significant.

## Results

### *In vitro* culture of embryonic epicardial cells

**Figure 1:**
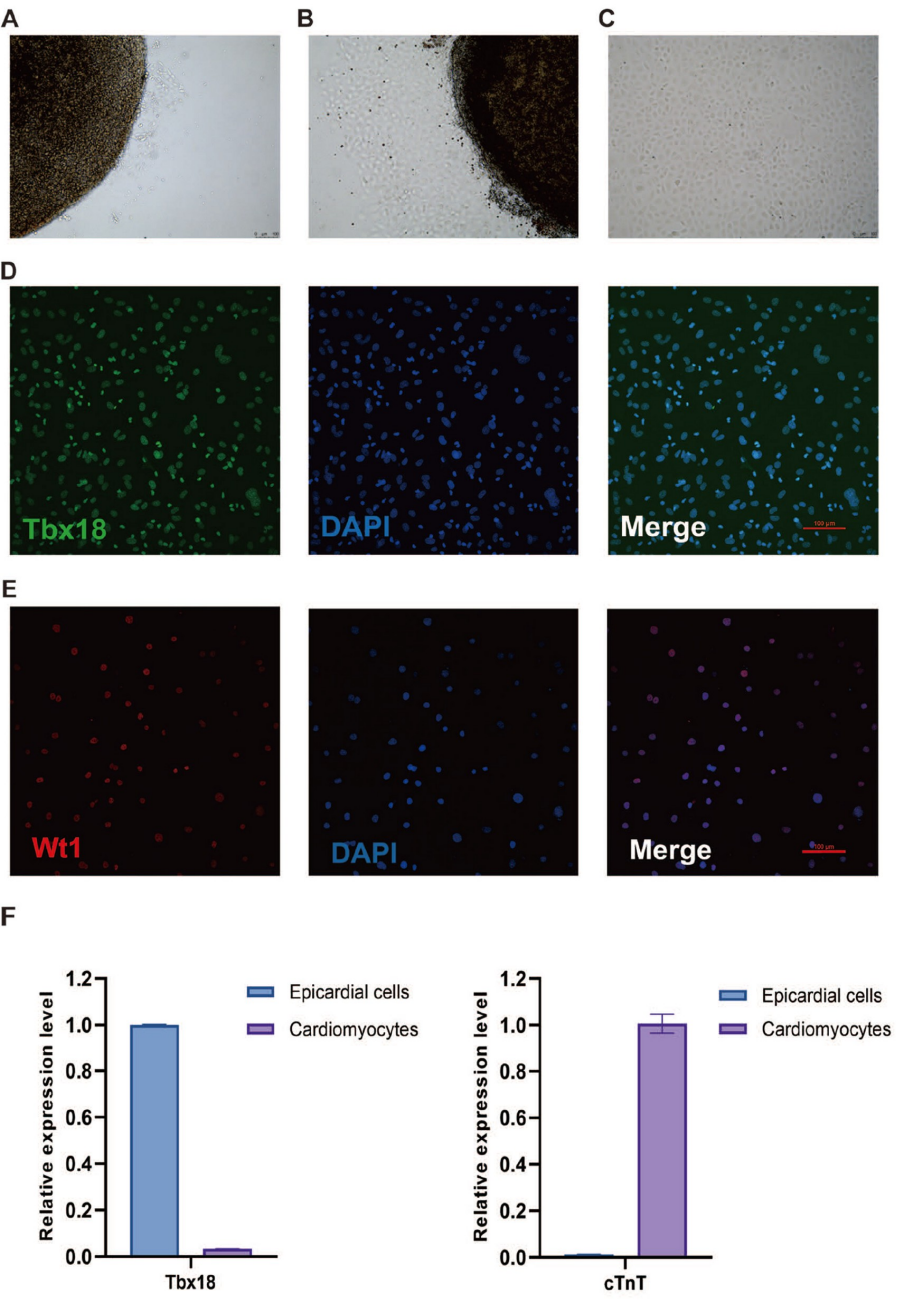
Figure 1 *In vitro* culture and validation of embryonic epicardial cells (A) After 24 h of culture, E12.5 ventricular tissue showed epicardial cells migrating from the tissue periphery. (B) After 48 h, more than ten layers of epicardial cells were observed emanating from the tissue edge. (C) Following the removal of ventricular tissue and an additional 48-h culture, the cells exhibited a “cobblestone” epithelial cell arrangement. Immunofluorescence staining results showing high levels of Tbx18 (D) and Wt1 (E) protein expression in the cultured cells. (F) qRT-PCR assays illustrating elevated Tbx18 mRNA levels in cultured cells compared to those in primary cardiomyocytes. In contrast, cardiomyocytes had high cTnT expression and minimal Tbx18 expression.

*In vitro* culture of E12.5 ventricular tissue for 24 h resulted in the migration of epicardial cells from the tissue periphery ( Figure 1A). After 48 h, we noted a multilayered emergence of these cells ( Figure 1B), which, post tissue removal, rapidly proliferated into a "cobblestone" arrangement after another 48 h ( Figure 1C). Further confirmation of the identity of these cells as embryonic epicardial cells was achieved via Tbx18 and Wt1 immunofluorescence staining respectively ( Figure 1D,E). Additionally, qPCR analysis verified the expression level of Tbx18 and minimal cardiac troponin T (cTnT) in embryonic epicardial cells, distinguishing them from primary cardiomyocytes ( Figure 1F). These results verified that the cells we cultivated with our technique are embryonic epicardial cells positive for Tbx18.

### TGF-β induces EMT and modulates miR-194-3p expression in epicardial cells

**Figure 2:**
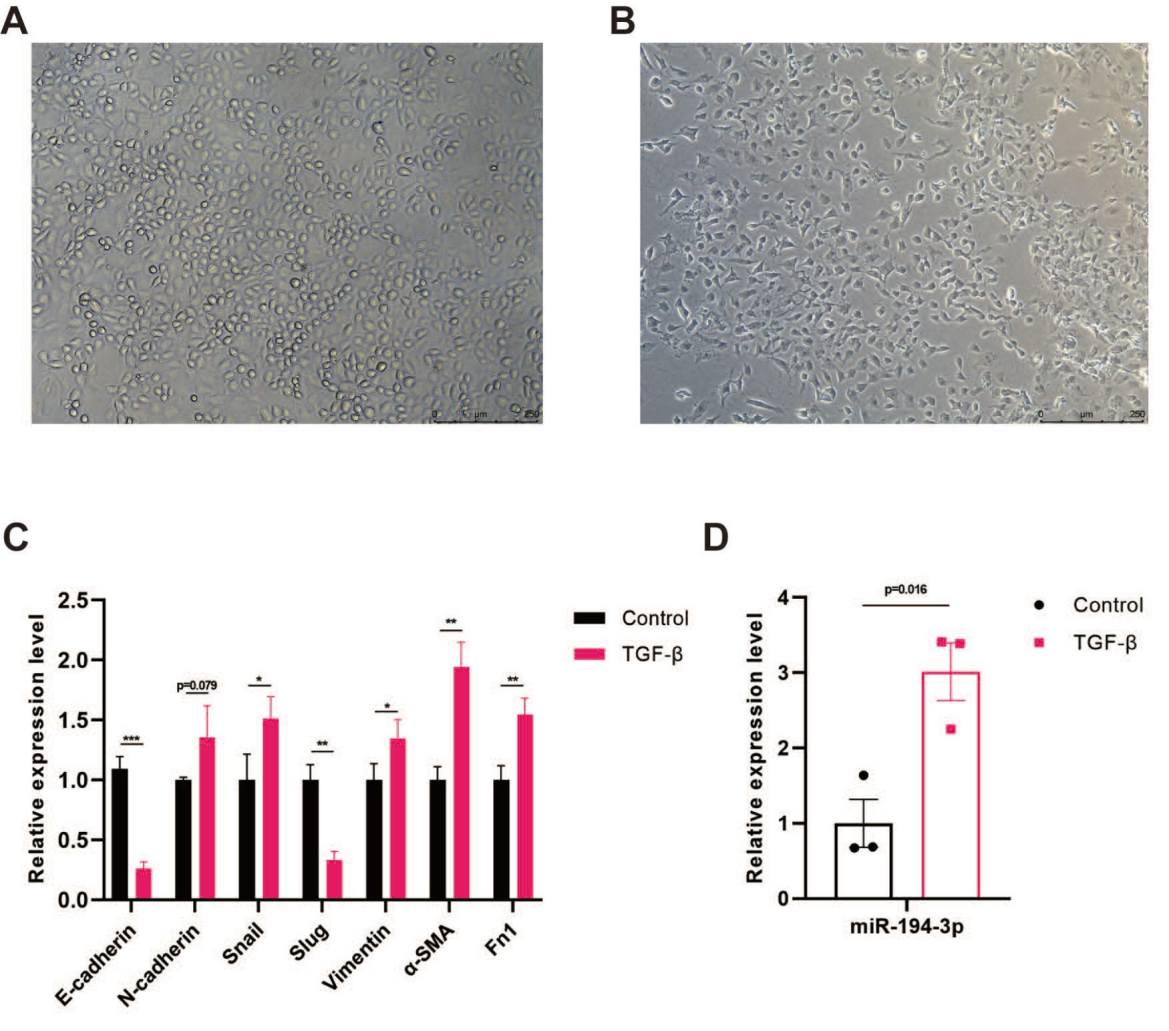
Figure 2 TGF- **β** induces EMT and modulates miR-194-3p expression in epicardial cells (A) Epicardial cell morphology of the control group. (B) Cell morphology of epicardial cells induced by 10 ng/mL TGF-β1 for 48 h. (C) qPCR was used to detect the alteration of EMT marker expression levels. *P<0.05, **P<0.01, ***P<0.001. (D) Quantitative analysis of miR-194-3p expression levels following TGF-β-induced EMT. TGF-β, transforming growth factor-β; α-SMA, α-smooth muscle actin; Fn1, fibronectin 1.

After treating epicardial cells with 10 ng/mL TGF-β1 for 48 h, a clear morphological shift was observed, with cells transitioning from an epithelial-like state ( Figure 2A) to a more spindle-shaped or irregular mesenchymal phenotype ( Figure 2B). The qPCR analysis showed TGF-β treatment decreased E-cadherin expression but elevated the levels of mesenchymal markers such as Snail, vimentin, Fn1, and α-SMA ( Figure 2C), indicating that TGF-β induced epithelial-to-mesenchymal transition in these cells. The expression of miR-194-3p was significantly increased after TGF-β-induced EMT in epicardial cells ( Figure 2D).

### miR-194-3p induces EMT process in epicardial cells

**Figure 3:**
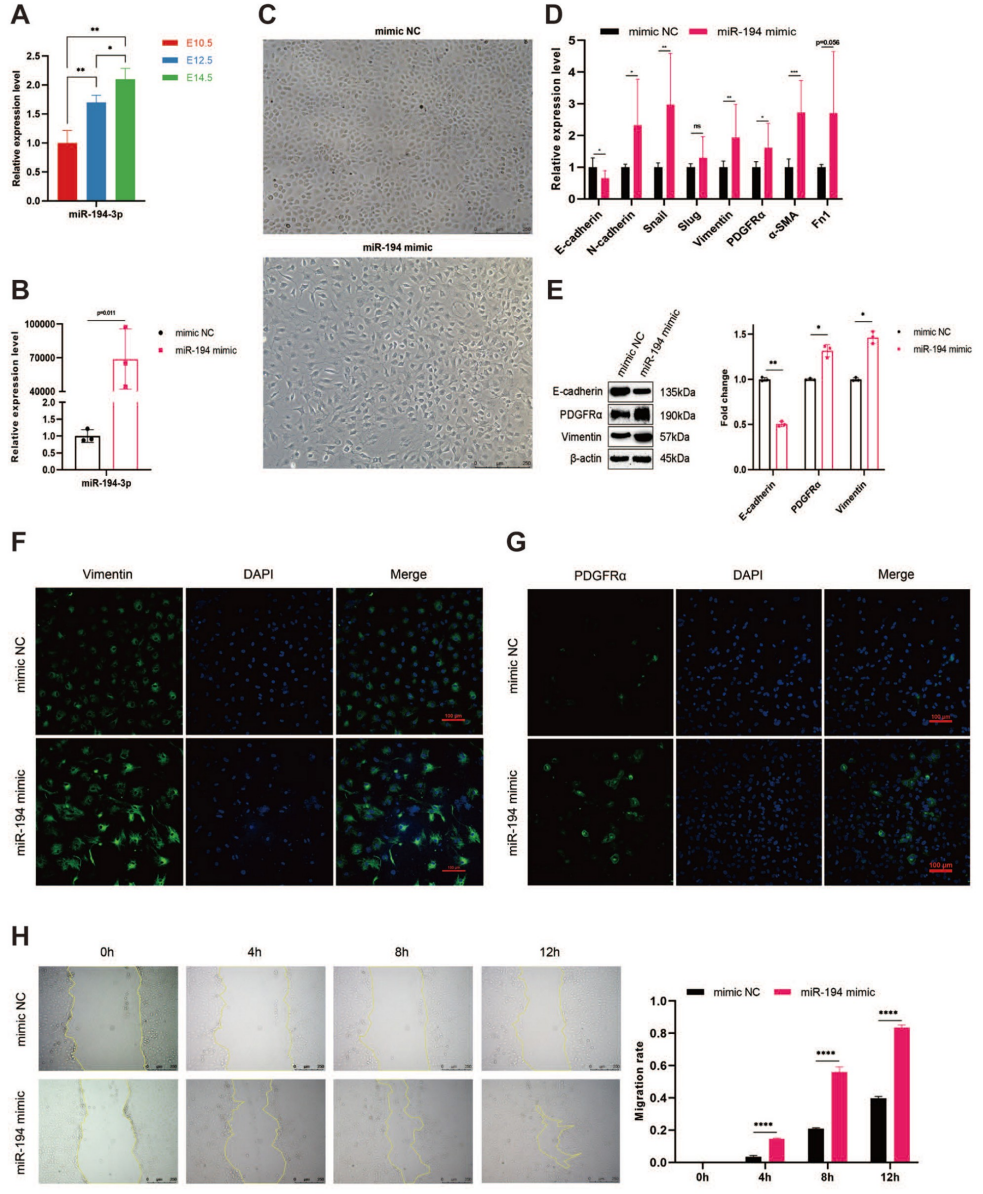
Figure 3 miR-194-3p promotes EMT in epicardial cells (A) qPCR analysis showing significant changes in miR-194-3p expression at different embryonic stages (E10.5, E12.5, and E14.5). n=3, *P<0.05, **P<0.01. (B) Validation of miR-194-3p overexpression after transfection. (C) Morphological changes to a mesenchymal phenotype. (D) qPCR results showed decreased E-cadherin level and increased levels of mesenchymal markers. *P<0.05, **P<0.01, ***P<0.001; ns, not significant. (E) Western blot analysis showing a reduction in E-cadherin expression and an increase in vimentin and PDGFRα expressions. n=3, *P<0.05, **P<0.01. (F,G) Enhanced vimentin and PDGFRα fluorescence in miR-194-3p-overexpressing cells. (H) Wound healing assay was used to detect cell migration after scratching for 0, 4, 8, or 12 h, respectively. n=3, ****P<0.0001. E, embryonic days

The expression of miR-194-3p at several embryonic stages, such as E10.5, E12.5, and E14.5, was subjected to qPCR analysis. The results showed significant alterations, suggesting that miR-194-3p is involved in the EMT process throughout these crucial periods of cardiac development ( Figure 3A). After transfecting miR-194-3p mimics into embryonic epicardial cells , the upregulation of miR-194-3p was confirmed ( Figure 3B), which led to a loss of epithelial arrangement, indicating a mesenchymal shift ( Figure 3C). qPCR results showed that the level of E-cadherin was decreased and the mesenchymal markers such as N-cadherin, Snail, and vimentin were increased ( Figure 3D). Western blot analysis further supported these findings, showing reduced E-cadherin and increased vimentin and PDGFRα levels ( Figure 3E and Supplementary Figure S1A‒D). Immunofluorescence revealed more vimentin and PDGFRα in miR-194-3p-overexpressing cells than in control cells ( Figure 3F,G). Furthermore, in the wound healing assays, these cells migrated more readily ( Figure 3H). In epicardial cells, miR-194-3p overexpression can generally induce EMT.

### Inhibition of miR-194-3p suppresses EMT in epicardial cells

**Figure 4:**
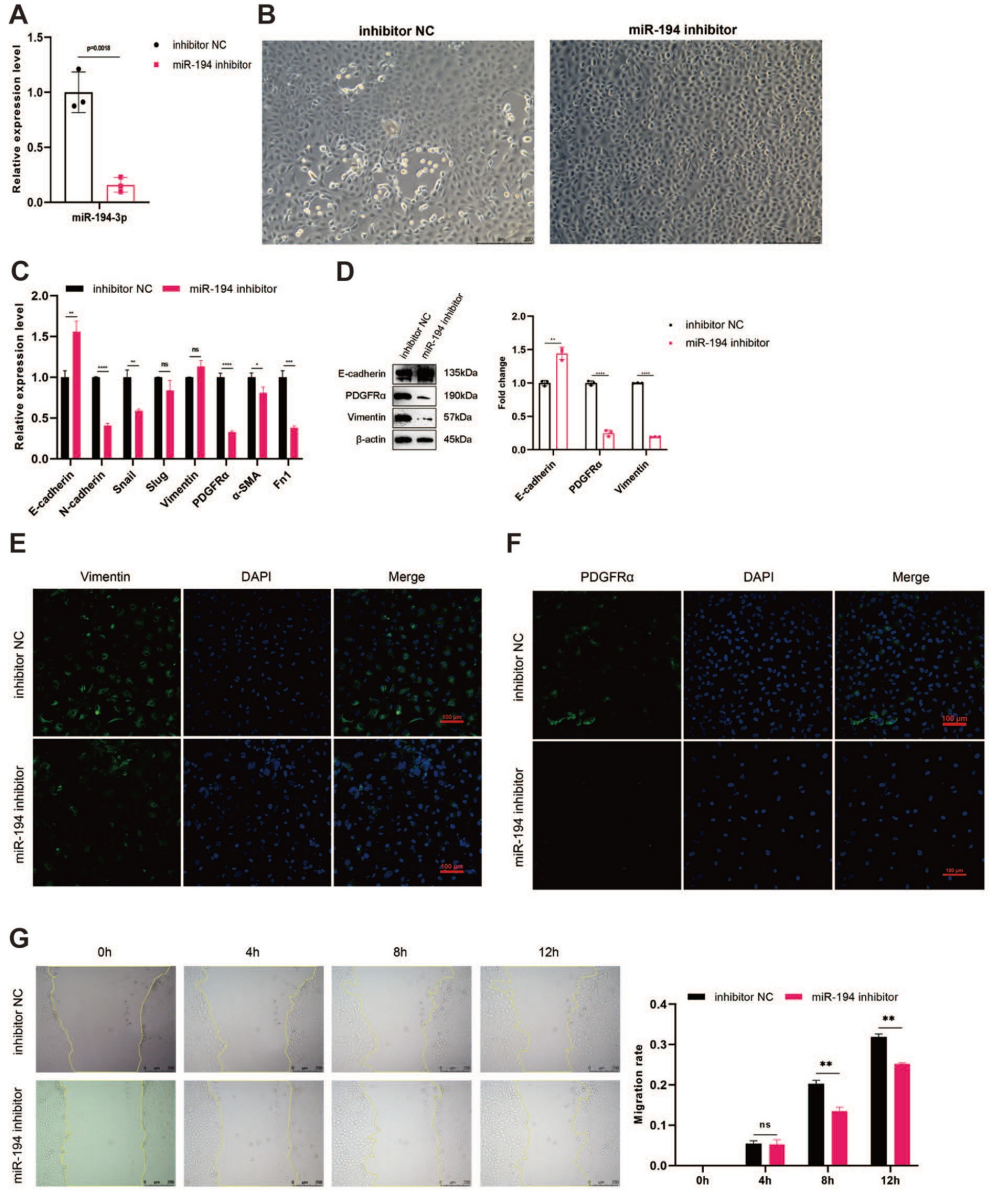
Figure 4 Inhibition of miR-194-3p suppresses EMT in epicardial cells (A) Decreased miR-194-3p expression post-inhibitor transfection. (B) Denser cellular arrangement upon miR-194-3p suppression. (C) Alteration of EMT markers detected by qPCR after the inhibition of miR-194. *P<0.05, **P<0.01, ***P<0.001, ****P<0.0001; ns, not significant. (D) Altered levels of EMT markers detected by western blot analysis after miR-194 inhibition. n=3, **P<0.01, ****P<0.0001. (E,F) Reduced fluorescence of vimentin and PDGFRα post miR-194-3p inhibition. (G) Wound healing assay was used to detect cell migration after scratching for 0, 4, 8, or 12 h, respectively. n=3, **P<0.01; ns, not significant.

We used inhibitors to limit the expression of miR-194-3p to investigate its inhibitory effect on epicardial cell EMT. After miR-194-3p inhibitor transfection, miR-194-3p expression was reduced significantly ( Figure 4A). These cells showed a denser arrangement ( Figure 4B), increased E-cadherin expression, and decreased expressions of mesenchymal markers, such as N-cadherin and Snail ( Figure 4C). Western blot analysis confirmed that the level of E-cadherin was increased and vimentin and PDGFRα levels were reduced ( Figure 4D and Supplementary Figure S2A‒D). Immunofluorescence staining results highlighted fewer vimentin and PDGFRα-expressing cells ( Figure 4E,F). The miR-194-3p-inhibited cells also showed reduced migration after 8 h, as revealed by wound healing assay ( Figure 4G). These results suggest that miR-194-3p inhibition hinders EMT in epicardial cells.

### Bioinformatics analysis and target prediction of miR-194-3p

**Figure 5:**
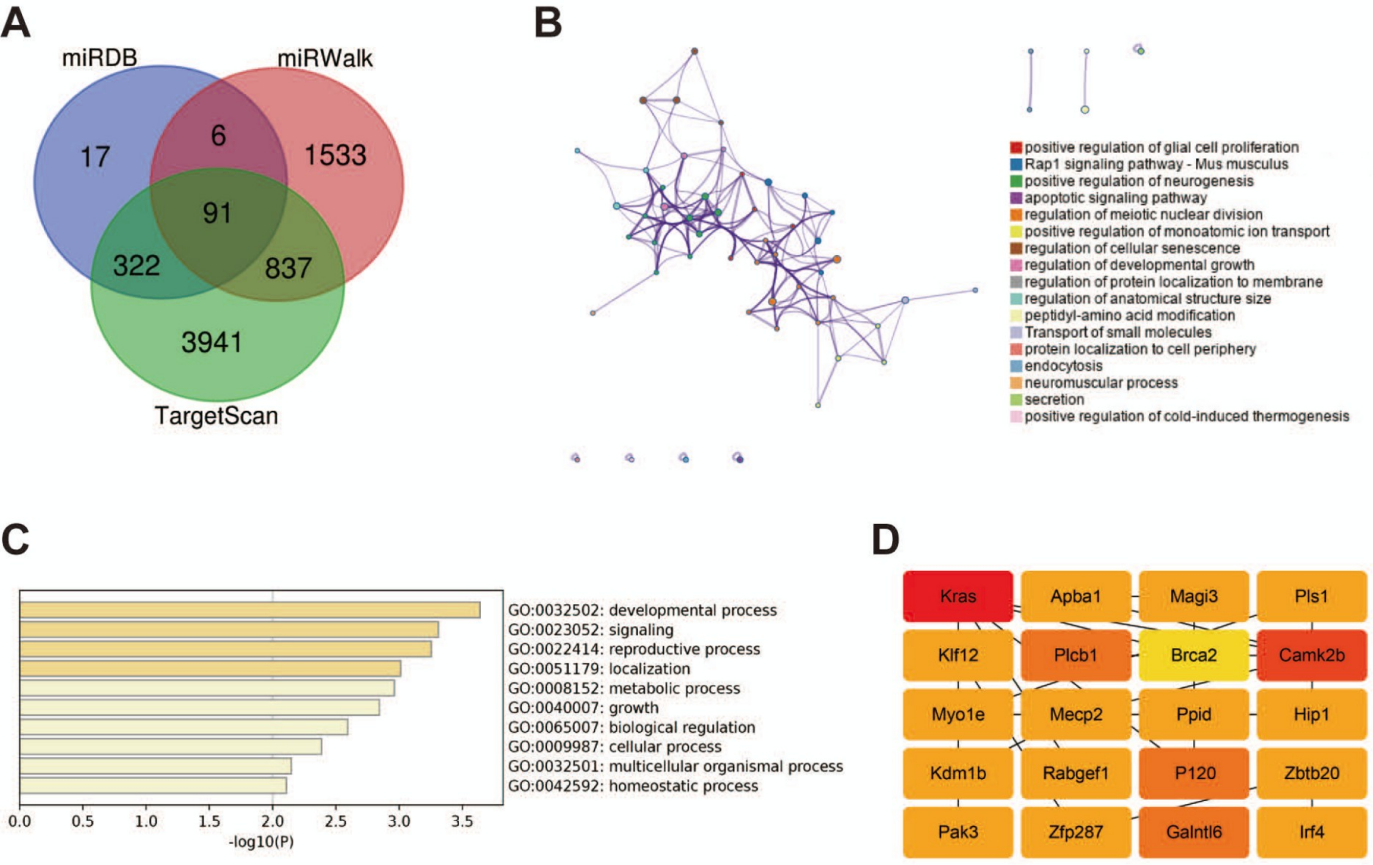
Figure 5 Bioinformatics prediction and analysis of miR-194-3p targets (A) Venn diagram depicting the overlap of target genes from three database predictions. (B) GO enrichment network for predicted targets; nodes represent specific functional ontology terms, with node size reflecting the number of genes within that term. (C) Enrichment analysis detailing the predicted biological processes associated with the target genes. (D) Hub genes from the predicted targets were identified using the CytoHubba app within Cytoscape; node color transitions from yellow to red based on hub gene scores.

We used TargetScan, miRWalk, and miRDB, three online databases in concert to identify the downstream target genes of miR-194-3p effectively. Ninety-one frequently predicted target genes were obtained by using a Venn diagram to show where the predictions from various databases intersected ( Figure 5A). Subsequent Gene Ontology (GO) analysis, performed using Metascape, revealed an enrichment of the predicted target genes in roles associated with the regulation of developmental growth, regulation of protein localization to the membrane, and secretion ( Figure 5B). Further analysis illustrated a pronounced enrichment in biological processes, including developmental processes, signaling, growth, and multicellular organismal processes ( Figure 5C). Additionally, hub genes among the predicted targets were identified based on the PPI network analysis using the CytoHubba app within the Cytoscape software ( Figure 5D).

### miR-194-3p regulates epicardial cell EMT via targeting p120/β-catenin

**Figure 6:**
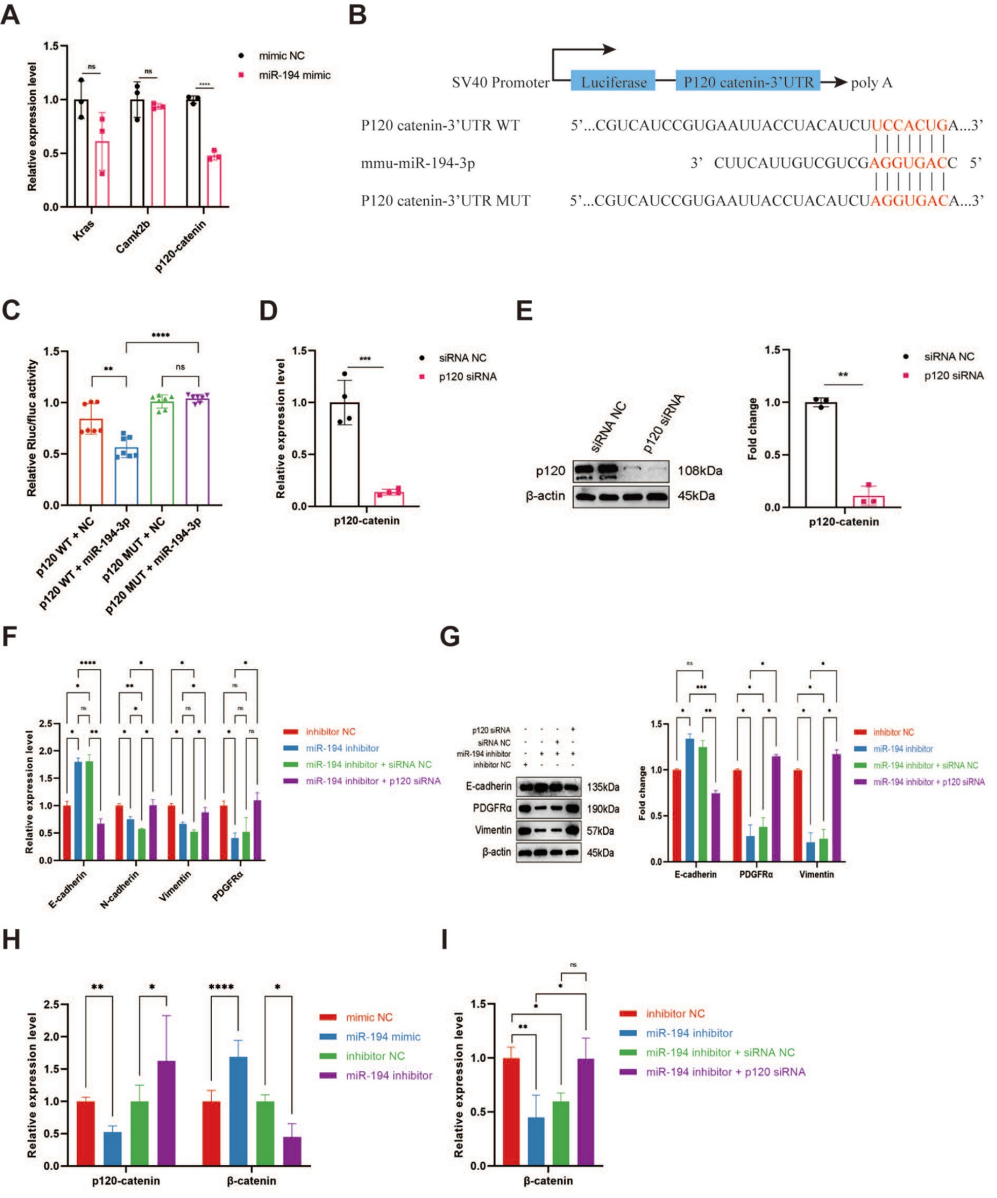
Figure 6 Regulation of epicardial cell EMT by miR-194-3p through the p120/β-catenin pathway (A) Altered expression of downstream predicted target genes detected by qPCR after overexpression of miR-194-3p. n=3, ****P<0.0001; ns, not significant. (B) Construction of wild-type and mutated 3′UTR variants of p120-catenin. (C) Decreased luciferase activity after co-transfection with the miR-194-3p mimic and the wild-type 3′UTR of p120-catenin, highlighting a direct interaction. n=7, *P<0.01, ****P<0.0001; ns, not significant. (D) qPCR assays showing the effect of p120-catenin siRNA on gene silencing. n=4, ***P<0.001. (E) Western blot anlaysis of the protein inhibition effect of p120-catenin siRNA. n=3, **P<0.01. (F) Rescue experiment showing the reversal of the EMT-suppressing phenotype caused by miR-194-3p inhibition. *P<0.05, **P<0.01, ****P<0.0001; ns, not significant. (G) Results consistent with those of the rescue experiment at the protein level. n=3, *P<0.05, **P<0.01, ***P<0.001; ns, not significant. (H) Altered p120-catenin and β-catenin levels upon modulation of miR-194-3p expression. *P<0.05, **P<0.01, ****P<0.0001. (I) The regulatory relationship between p120-catenin and β-catenin was confirmed by a rescue experiment. *P<0.05, **P<0.01; ns, not significant.

In the context of the previously elucidated potential target genes, we selected three prominent candidates: Kras, Camk2b, and p120-catenin. Notably, when miR-194-3p was overexpressed, there was a significant decline in *p120-catenin* mRNA expression. On the other hand, there was little change in the levels of Kras and Camk2b, suggesting that miR-194-3p and p120-catenin may have a regulatory relationship ( Figure 6A). Luciferase reporter assay confirmed that miR-194-3p and p120-catenin have a direct interaction by using wild-type and mutant constructs of the *p120-catenin* 3′UTR( Figure 6B,C). The expression of p120-catenin was significantly inhibited at both the mRNA and protein levels by *p120-catenin* siRNA ( Figure 6D,E and Supplementary Figure S3A,B). Remarkably, rescue experiments demonstrated that silencing of *p120-catenin* with siRNA could reverse the EMT inhibition caused by the miR-194-3p inhibitor, underscoring p120-catenin’s critical role in miR-194-3p-mediated epicardial cell EMT ( Figure 6F,G and Supplementary Figure S4A‒D).

To confirm the role of p120-catenin in cell adhesion via E-cadherin, we further explored its potential interaction with β-catenin. Overexpression of miR-194-3p led to reduced p120-catenin level and elevated β-catenin level, while its inhibition showed the opposite effect ( Figure 6H). Furthermore, the reduced β-catenin expression induced by the miR-194 inhibitor was rescued by *p120-catenin* siRNA ( Figure 6I), confirming that p120-catenin acts upstream of β-catenin. All of these results implied the possibility that miR-194-3p stimulates epicardial cell EMT through p120-catenin inhibition, which in turn activates β-catenin.

## Discussion

The mesothelial layer of the heart, known as the epicardium, has long been acknowledged as a special structure that is crucial for both endogenous regenerative repair and cardiac growth. Over the past decade, abundant research has confirmed the involvement of epicardial cells in vascular system stabilization [ 28, 29], conduction system formation [30], and myocardial compaction processes [ 31, 32]. The consequences of disrupted epicardial formation, such as thinning of the myocardial wall and coronary artery defects, underscore the importance of the epicardium in heart development [ 33, 34]. The function of microRNAs in controlling the development of the embryonic heart is still poorly understood today. Accumulating evidence suggests that a restricted set of microRNAs might regulate Twist [35] and/or Zeb1/Zeb2 [36] during development and/or tissue homeostasis. In the process of endocardial cushion formation, three microRNAs, namely miR-23, miR-126, and miR-199, have been implicated in EMT [ 37– 39], all of which act as suppressors. The conserved functional characteristics of these genes across species such as mice, chickens, and zebrafish further bolster the widespread evolutionary conservation theory. Regrettably, our comprehension of microRNA regulation during epicardial-to-mesenchymal transition remains markedly deficient [ 40– 42]. It is noteworthy that reduced epicardial development has been associated with the loss of the Dicer enzyme, which is essential for microRNA processing but does not negatively impact EMT [42].

The role of miR-194-3p in the development of the embryonic heart and its regulatory effects on EMT are documented for the first time in our current investigation. Research on miR-194-3p has been limited. In specific cancer contexts, miR-194-3p primarily mitigates the TGF-β-induced EMT promotion [43] and inhibits cell migration and invasion [ 21, 22, 44– 47]. Intriguingly, our findings, veriying miR-194-3p as a promoter of epicardial cell EMT and migration, are in stark contrast to these earlier observations. This discrepancy may stem from the different contexts of carcinogenesis versus embryonic development. Our initial research suggested a potential link between autophagy and the developmental differentiation of epicardial cells [48]. Recent insights suggested the potential regulatory role of miR-194-3p via autophagy [49], yet a deeper exploration into its precise mechanisms is warranted. In addition to the modulation of EMT-related signaling pathways and key transcriptional regulators by microRNAs, emerging studies have spotlighted the direct regulatory effect of microRNAs on cell-cell adhesion and cytoskeletal proteins. For instance, miR-122, miR-24, and miR-1291 have been shown to directly target RhoA expression [ 50– 52], while miR-22 impacts actin filament expression [53], thereby providing cues for cytoskeletal remodeling during epithelial-to-mesenchymal transition. It is known that a number of microRNAs affect the expressions of claudins and cadherins, which are essential for cell-cell junctions. Notably, both miR-10b and miR-214 have been documented to directly target the 3′ UTR of *E-cadherin* [ 54, 55], while indirect regulation of E-cadherin has been attributed to numerous microRNAs, including the miR-200 family [ 56– 59]. Furthermore, miR-27a has been identified to regulate vascular endothelial-cadherin directly [60], while miR-199a has been implicated in modulating the expression of N-cadherin [61]. Furthermore, miR-155 and miR-30a, two microRNAs, directly target Claudin 1 and Claudin 5, respectively [ 62, 63].

The cooperative utilization of three online databases identified 91 putative downstream targets for miR-194-3p, highlighting its potentially complex function in the development of the embryonic heart. Notably, the PPI analysis spotlighted Kras, Camk2b, and p120-catenin as the hub genes, which intriguingly have not been previously reported in the context of embryonic epicardial cell EMT. The involvement of these genes could introduce a novel layer of complexity and regulation in the EMT process, warranting further investigation to elucidate their specific roles and interactions mediated by miR-194-3p. The adhesive junctions of epithelial cells play an indispensable role in their growth and developmental trajectory. These junctions serve as the hubs for the interactions between cells and are essential regulators of cell migration, proliferation, polarization, and survival [ 64, 65]. Within the cellular milieu, E-cadherin engages in the interactions with members of the catenin family, among which p120-catenin is crucial for the stability of cadherins. Research using animal models and cell culture has indicated that E-cadherin serves as a tumor suppressor and barrier against invasion, which is consistent with its importance in normal development and homeostasis.

Intracellularly, E-cadherin interacts with p120 through its juxta membrane domain (JMD) and with β-catenin via its catenin-binding domain [66]. β-Catenin, in turn, links E-cadherin to the actin cytoskeleton through interactions with α-catenin. JMD targets E-cadherin for endocytosis and proteasomal breakdown through endocytic processes and docking sites for the ubiquitin ligase Hakai [65]. By shielding these sites, p120 emerges as a principal regulator of E-cadherin stability [ 67‒ 69]. In our study, the observed effects of miR-194-3p overexpression on epicardial cell EMT and cell adhesiveness align with the reduced level of p120-catenin, an integral player in cell adhesion signaling. This relationship between miR-194-3p and p120-catenin is intriguing, as it appears to initiate a cascade of changes, notably in β-catenin dynamics. A compensatory increase in signaling molecules, such as β-catenin, which are known to play roles in the advancement of epithelial-mesenchymal transition, may be triggered by the loss of cell adhesion, as indicated by the increase in free β-catenin that follows the decrease in p120-catenin. Our findings contribute to a understanding of how microRNAs influence cell behavior by modulating key adhesion molecules. However, a complete understanding of the specific molecular relationship between β-catenin dynamics and decreased p120-catenin level is still pending. Future studies should aim to directly quantify the binding between p120-catenin and β-catenin to unravel this complex regulatory relationship, providing valuable insights into the molecular intricacies of EMT, not only in the context of cardiac development but also in pathological states where EMT plays a critical role.

In conclusion, our study sheds light on the complex functions of the cytoskeleton, developmental signaling pathways, and microRNA-associated cell junctions while offering a novel understanding of the expression of miR-194-3p during epicardial development. However, the emphasis on miR-194-3p may obscure other influential microRNAs, and while our findings are rooted in *ex vivo* models, *in vivo* validation remains imperative. This basic information not only opens up new research directions for the study of noncoding RNAs in general and cardiac development in particular, but it also points to possible treatment approaches for heart diseases.

## Supporting information

519Supplementary_data

## Supplementary Data

Supplementary data is available at *Acta Biochimica et Biophysica Sinica* online.
