# Supplementary material for: miR-194-3p regulates epithelial-mesenchymal transition in embryonic epicardial cells via p120/β-catenin signaling: miR-194-3p regulates EMT via p120/β-catenin
Source: Acta Biochim Biophys Sin (Shanghai). 2024 Apr 26;56(5):717–29. doi: 10.3724/abbs.2024051 (PMC11381220; doi:10.3724/abbs.2024051)
Supplement: 519Supplementary_data [file 519Supplementary_data.docx]

# Supplementary Material

##
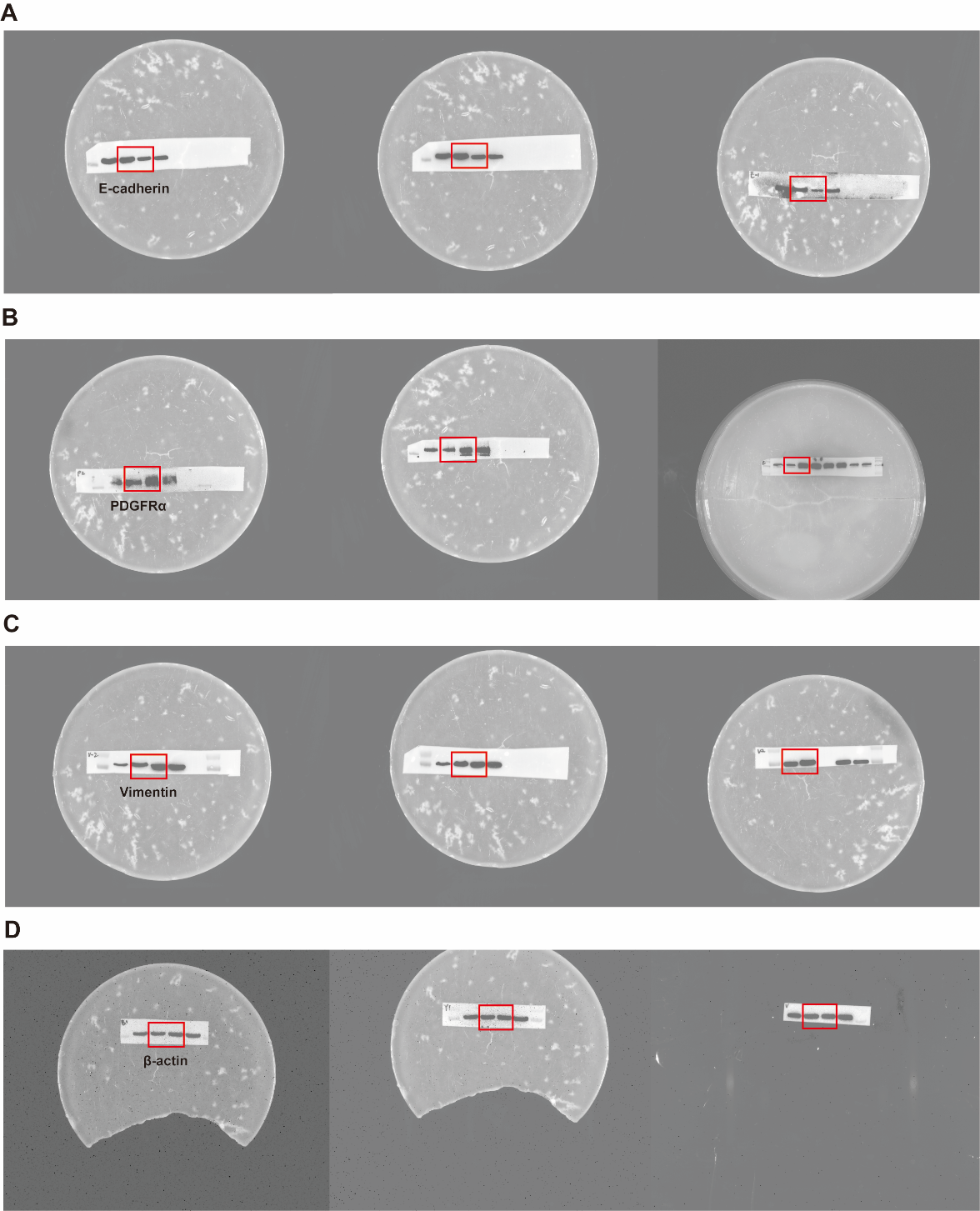


**Supplementary Figure S1. Uncropped full-length blots corresponding to Figure 3E in the main text** (A) E-cadherin, (B) PDGFRα, (C) vimentin, and (D) β-actin. The regions cropped and used in the main text are indicated by red boxes.


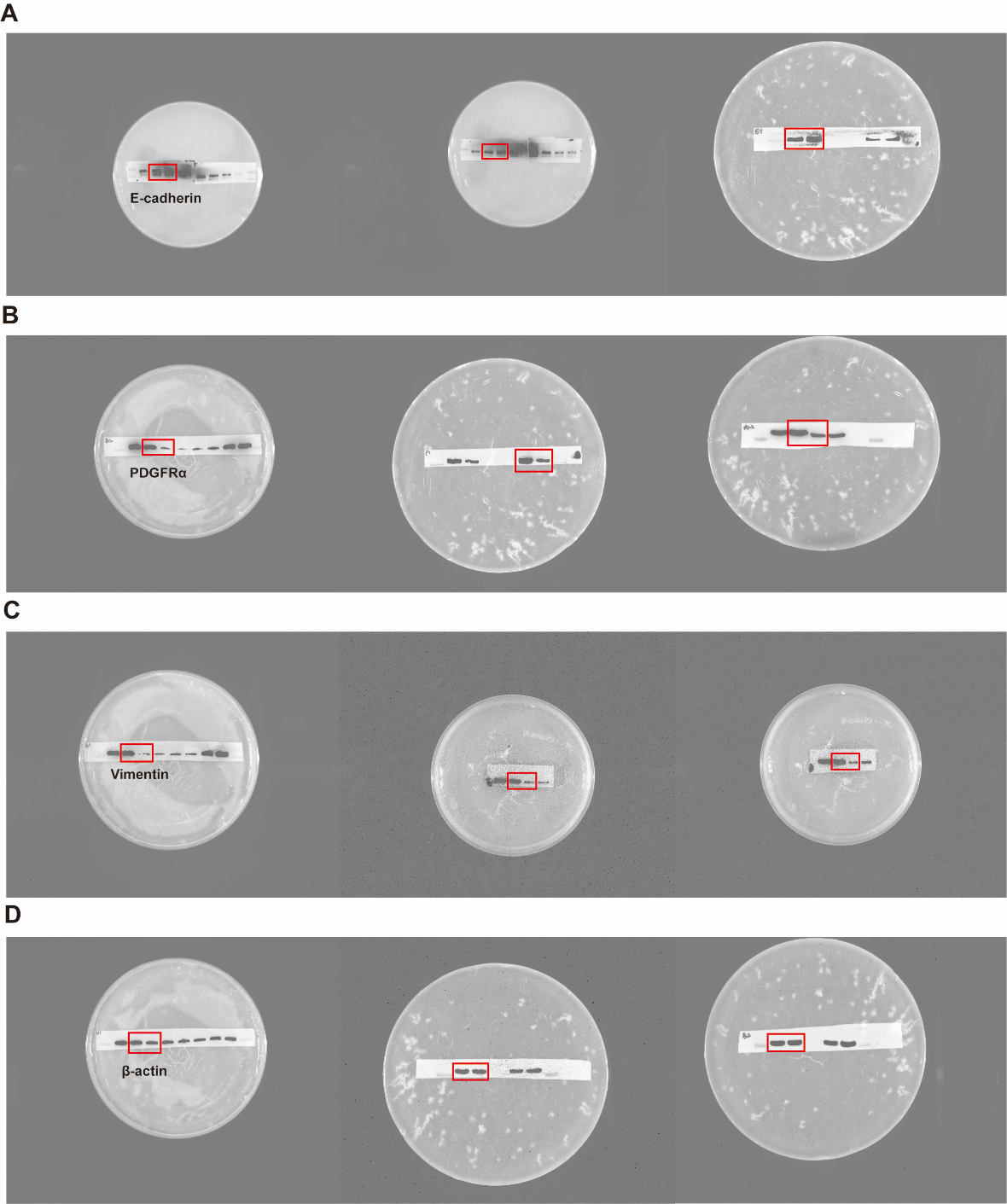


**Supplementary Figure S2. Uncropped full-length blots corresponding to Figure 4D in the main text** (A) E-cadherin, (B) PDGFRα, (C) vimentin, and (D) β-actin. The regions cropped and used in the main text are indicated by red boxes.


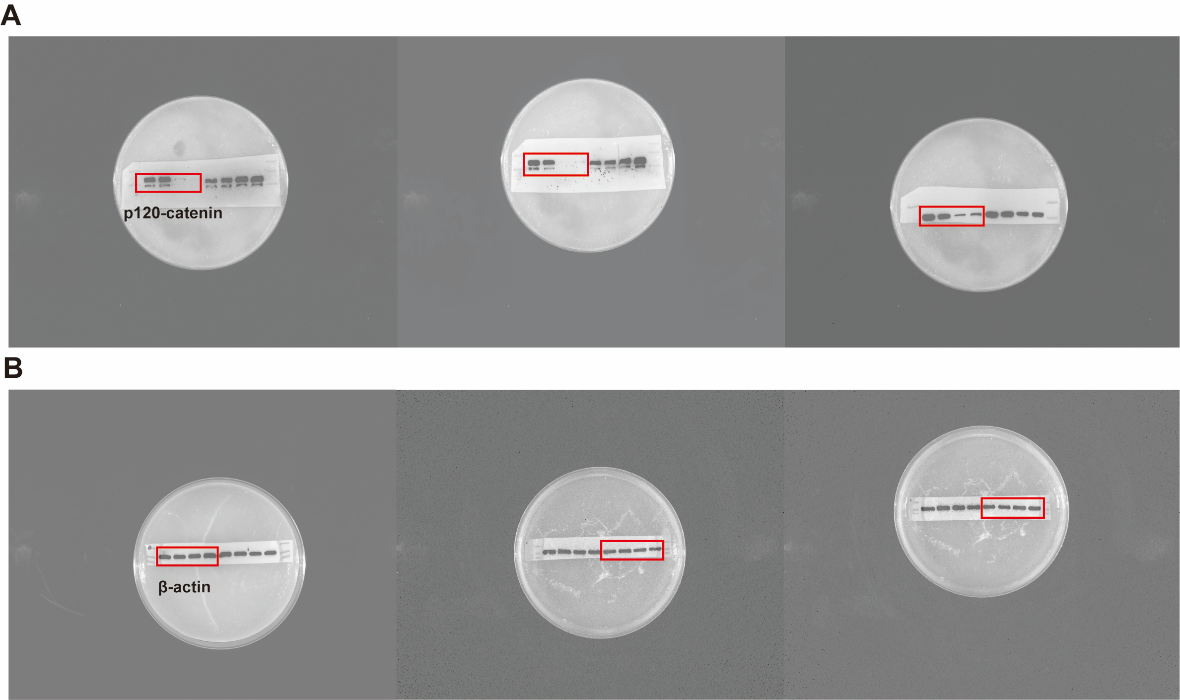


**Supplementary Figure S3. Uncropped full-length blots corresponding to Figure 6E in the main text** (A) p120-catenin, (B) β-actin. The regions cropped and used in the main text are indicated by red boxes.


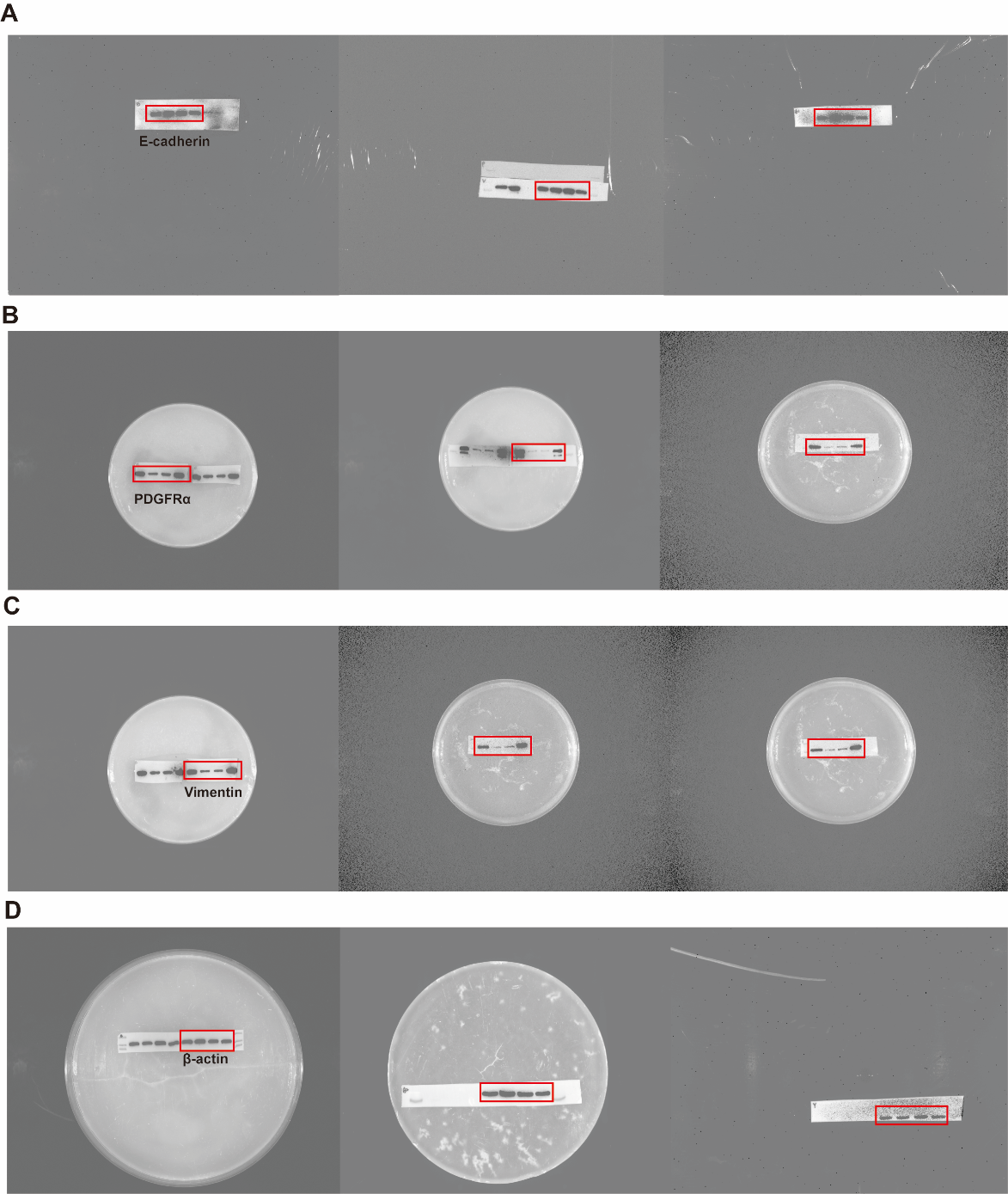


**Supplementary Figure S4. Uncropped full-length blots corresponding to Figure 6G in the main text** (A) E-cadherin, (B) PDGFRα, (C) vimentin, and (D) β-actin. The regions cropped and used in the main text are indicated by red boxes.
